# Clinical serum lipidomic profiling revealed potential lipid biomarkers for early diabetic retinopathy

**DOI:** 10.1038/s41598-024-66157-z

**Published:** 2024-07-02

**Authors:** Wen Sun, Miaomiao Su, Lvyun Zhuang, Yujun Ding, Qianhui Zhang, Daizhu Lyu

**Affiliations:** 1 Ophthalmology, Hainan Provincial Hospital of Traditional Chinese Medicine, Haikou, 571100 China; 2 Ophthalmology, Hainan Medical College Affiliated Chinese Medicine Hospital, Haikou, 571100 China; 3 Analysis and Testing Center, Chinese Academy of Tropical Agricultural Sciences, Haikou, 571100 China

**Keywords:** Diabetic retinopathy, Non-proliferative diabetic retinopathy, Lipid markers, Lipidomics, Targeted proteomics, Biochemistry, Biological techniques, Computational biology and bioinformatics, Biomarkers, Diseases

## Abstract

Diabetic retinopathy (DR) is a serious complication of diabetes featuring abnormal lipid metabolism. However, the specific lipid molecules associated with onset and progression remain unclear. We used a broad-targeted lipidomics approach to assess the lipid changes that occur before the proliferative retinopathy stage and to identify novel lipid biomarkers to distinguish between patients without DR (NDR) and with non-proliferative DR (NPDR). Targeted lipomics analysis was carried out on serum samples from patients with type I diabetes, including 20 NDRs and 20 NPDRs. The results showed that compared with the NDR group, 102 lipids in the NPDR group showed specific expressions. Four lipid metabolites including TAG58:2-FA18:1 were obtained using the Least Absolute Shrink And Selection Operator (LASSO) and Support Vector Machine Recursive Feature Elimination (SVM-RFE) methods. The four-lipid combination diagnostic models showed good predictive ability in both the discovery and validation sets, and were able to distinguish between NDR patients and NPDR patients. The identified lipid markers significantly improved diagnostic accuracy within the NPDR group. Our findings help to better understand the complexity and individual differences of DR lipid metabolism.

## Introduction

Diabetic retinopathy (DR) is a serious microvascular complication of diabetes mellitus (DM) and one of the main causes of adult blindness. This condition, along with threatening the quality of life of patients with DM, carries a significant economic burden to social medical security^1–3^. In 2010, the global prevalence of DR was estimated to be 126 million. Without intervention, this number is expected to increase to 119 million by 2030^4^. Yau et al., performed a comprehensive analysis of 35 population-based studies from around the world, and their data showed that the estimated prevalence of DR among patients with DM was 34.6% (9.3 million individuals), with sight-threatening DR accounting for 10.2% cases^5,6^. Therefore, early screening and diagnosis are of utmost importance for improving the prognosis of DR.

DR is classified into two stages: non-proliferative diabetic retinopathy (NPDR) and proliferative diabetic retinopathy (PDR). Patients with NPDR have microaneurysms, retinal hemorrhage, retinal thickening (edema), and exudation, thus leading to decreased central vision. Patients with PDR experience neovascularization of the optic disc, retina, iris, and filtration angle, thus leading to tractional retinal detachment, neovascular glaucoma, and blindness^7^. However, the pathogenesis of DR is not fully understood. Studies have found that the dysfunction of inflamed neurons, abnormal gene expression, epigenetic changes, disrupted signaling pathways, and metabolic products are all involved in the process of DR^8–10^.

In several patients, intensified metabolic control is a highly effective method for controlling DR and other diabetes-related complications. Therefore, an early diagnosis can prevent and intervene, thereby reducing its incidence and delaying disease progression. Several studies have attempted to identify predictive and diagnostic molecular markers of DR. Using serum transcriptome analysis of 20 PDR and 20 NPDR patients, Pan et al. found that CCDC144NL, DYX1C1, and other genes could be used to predict the occurrence of PDR, with a sensitivity and specificity of over 90%^11^. Liu et al., found significant differences in the expression of lysophosphatidic acid receptor 3 (LPAR3) and calponin (CNN1) in the peripheral blood leukocytes of patients with DR and DM, thereby suggesting that LPAR3 and CNN1 could be used as biomarkers for DR diagnosis. Chee et al.^12^, used proteomics to analyze the salivary proteins of NPDR, without DR (NDR), and PDR patients with T2DM and found 119 differentially expressed proteins. Among them, the proteins with the most increased expression in the PDR group were defense and metabolic proteins related to the immune inflammatory response, thus indicating that these may be biomarkers for NPDR progression to PDR. Balaiya et al.^13^ used proteomics to analyze the vitreous humor of patients with PDR and patients with preretinal membranes or macular holes and found 16 unique proteins in the vitreous humor of patients with PDR that can be used as biomarkers. Curovic et al.^14^ evaluated serum lipid metabolites in patients with DR of different grades and found that 3,4-dihydroxybutyric acid in the serum was a biomarker for DR progression.

In recent years, an increasing number of studies have focused on the relationship between lipid metabolism disorders and the pathogenesis of diabetes^15–18^. Additionally, retinal lipids play crucial roles in retinal function and disease. Protein kinase C (PKC) is believed to play an important role in the development of diabetic complications, and PKC stimulation in diabetes is mediated solely by the excessive production of diacylglycerol rich in palmitic and oleic acid. Changes in blood glucose concentration and free fatty acid levels are closely related, and these changes, apart from oxidative stress, may cause abnormal PKC activation in diabetes. It is unclear whether the decrease of polyunsaturated fatty acid composition in diabetes patients is due to the increase of lipid peroxidation or the change of lipid synthesis and circulation.The specific lipid molecules associated with onset and progression of DR remain unclear^19,20^. Therefore, this study aimed to use lipid metabolomics to detect potential lipid biomarkers for diagnosis of early DR using clinical samples. Lipidomics utilizes mass spectrometry to analyze a large number of metabolites with high throughput. Compared with traditional single biomarker methods, this research method can obtain comprehensive information on lipid metabolism, as well as allow for a more comprehensive understanding of the biological effects of various metabolites. We used targeted lipidomics based on ultrahigh-performance liquid chromatography-triple quadrupole mass spectrometry (UHPLC-MS/MS). Further, we comprehensively detected and compared the differences in serum lipid profiles between healthy controls (NDR) and NPDR groups and selected biomarkers with effective diagnostic performance for DR using univariate and multivariate statistical analyses and receiver operating characteristic (ROC) analysis. Lastly, we verified the differential lipid molecules in an independent case–control study consisting of 11 NDR and 11 NPDR patients. Our findings may provide a reference for the early screening and diagnosis of DR. Currently, diabetic retinopathy is primarily diagnosed using fundus photography, a method that can only detect the condition once retinal changes, such as hemorrhages, have occurred. This limitation is not conducive to early intervention and treatment of diabetic retinopathy (DR). The goal of our study is to explore new methods for the early diagnosis of diabetic retinopathy, providing new avenues for the early detection and prevention of this condition.

## Materials and methods

### Materials and preservation

The Ethics Committee of the Hainan Provincial Hospital of Traditional Chinese Medicine approved this study (reference number: 012/012). Han Chinese patients with T2DM were contacted and included in the study. The patients were between 35 and 75 years of age and were treated at the Hainan Provincial Hospital of Traditional Chinese Medicine. Patients who agreed to participate in the study were recruited. Patients who reported human immunodeficiency virus infection and other chronic diseases, such as tuberculosis, severe hypertension, thyroid disease, heart disease, kidney failure, arrhythmia, liver and kidney dysfunction, tumors, mental illnesses, pregnancy, lactation, and infectious and congenital diseases, were excluded from the study. Patients consuming multiple vitamins or statins were also excluded. Patients underwent clinical examination and selected participants underwent retinopathy screening, with further confirmation and documentation of the retina using a high-resolution fundus camera. The authors confirm that all methods were carried out in accordance with relevant guidelines and regulations. DR was classified according to the Early Treatment Diabetic Retinopathy Study. Patients with T2DM, with or without non-proliferative retinopathy, were included in the study. A total of 62 participants were recruited and subdivided into NDR (31) and NPDR (31) groups. 20 mL of fasting blood was collected in vacuum tubes. Plasma was separated from samples within 3 h of collection, and serum samples were stored at − 80 °C.

### Instruments and reagents

Ultra-high performance liquid chromatography-triple quadrupole tandem mass spectrometry (Triple QNPDRd 6500+AB SCIEX), liquid chromatography column Kinetex C18 (2.6 μm 2.1 × 100 mm Guangzhou Fenomei Scientific Instruments Co. Ltd.), one-ten-thousand electronic balance AL204 (Shanghai Mettler Toledo Instrument Co., Ltd.), floor-standing high-speed freezing centrifuge (CR22N Hitachi, Japan), ultra-pure water system Milli-Q Integral 3 (MILLIPORE, U.S.A.); nitrogen blowing instrument N-EVAP (U.S.A. Organomation, Inc.), thermostatic water bath HH-WO (Shanghai Niobio Instrument Co., Ltd.), CNC ultrasonic cleaner KQ-500DE (Kunshan Ultrasonic Instrument Co., Ltd.), acetonitrile, methanol (chromatographic purity, Merck Group Inc., USA), formic acid (mass spectrometry purity, Thermo Fisher Scientific, USA).

### Total lipid extraction

The serum was thawed on ice. 400 μL of serum was added to a 2 mL tube, with 1 mL of lipid extraction solution and an internal standard mixture. The mixture was vortexed for 2 min and sonicated for 10 min in a 4 ℃ water bath. Then, 500 μL of water was added and vortexed for 1 min, followed by centrifugation at 15,000 rpm for 10 min. The supernatant (500 μL) was collected and dried under nitrogen gas. The residue was re-dissolved in 100 μL of mobile phase B and vortexed for 1 min. The sample was then centrifuged at 14,000 g for 15 min at 4 ℃ and sonicated for 10 min in a 4 ℃ water bath. The sample was then cooled at – 20 ℃ for 1 h and centrifuged at 15,000 rpm for 10 min (r = 9 cm). The supernatant was collected and analyzed using mass spectrometry. 10 μL of each sample supernatant was added to a 2 mL tube and swirled, and 150 μL was used to prepare a sample quality control. Methanol (150 μL) was used as a blank control.

### UPLC-MS/MS analysis

Using UPLC-MS/MS (AB SCIEX, USA) as the platform for metabolite separation and detection, differences in metabolites between the NPDR and NDR groups were evaluated, and data were collected in positive and negative ion modes. A CSH C18 column (2.6 μm; 2.1 × 100 mm, Kinetex C18) and Luna NH2 column (3 μm; 2.0 × 100 mm) were used. The positive ion source mass spectrometry conditions were as follows: ion spray voltage (ISVF) was 5200 V, and ion source temperature was 350 °C. The negative ion source mass spectrometry conditions were as follows: ISVF was − 4500 V, and ion source temperature was 350 °C. Multiple reaction monitoring and triple quadrupole mass spectrometry were used for the targeted analysis of lipid group data, and SCIEX OS was used for peak identification and filtering.

Chromatographic method for the positive ion source: mobile phase A, water:methanol:acetonitrile (7 mM ammonium acetate, 1:1:1, v/v); mobile phase B, isopropanol (7 mM ammonium acetate); flow rate 0.3 mL/min; injection volume 1 μL; column temperature 45 °C; and gradient elution. Chromatographic method for the negative ion source: mobile phase A, isopropanol:acetonitrile (5 mM ammonium acetate, 7/93, v/v); mobile phase B, water: acetonitrile (2 mM ammonium acetate, 1:1, v/v); and gradient elution. The elution was performed using the following gradient elution conditions: For the positive source, 0–1.5 min, holding 20% B and 80% A; 1.5–3 min, increasing to 40% B and reducing to 60% A; 3–13 min, increasing to 60% B and reducing to 40% A; 13–13.1 min, increasing to 98% B and reducing to 2% A; and 13.1–17 min, decreasing to 20% B and increasing to 80% A. For the negative source, 0–2 min, holding 100% A; 2.01–7 min, increasing to 50% B and reducing to 50% A; 11–11.5 min, increasing to 70% and reducing to 30% A; 11.5–12.5 min, increasing to 100% B; 12.5–15 min, maintaining at 100% B; 15–15.1 min, decreasing to 0% B and increasing to 100% A; and 15.1–17 min, maintaining at 100% A.

### Data processing and statistical analyses

MultiQuant software was used to perform chromatographic peak review and peak area integration analysis of the mass spectrometry files of the samples. Each chromatographic peak represented a lipid metabolite, and the area under the peak corresponded to its relative abundance. This enabled the analysis and comparison of different lipids in serum samples. The significance of differences between datasets from different groups was defined using analysis of variance and Tukey’s test, with p < 0.05 indicating significant differences. OPLS-DA was performed on lipidomic data using SIMCA 14.1, and VIP values were calculated. Permutation tests were performed (200 permutations) to avoid overfitting. Screening for differential lipid metabolites using a combination of P values and multiple changes (FC) was performed using non parametric tests. Following this, differential lipid metabolites were screened using VIP ≥ 1, FC > 1.5, or FC < 0.66 and P < 0.05 as standards. The Kyoto Encyclopedia of Genes and Genomes (KEGG) was used to annotate the functions of enriched differential lipid metabolites, and the Least Absolute Shrinkage Selection Operator (LASSO) regression and Support Vector Machine (SVM) methods were used to further select features and construct a diagnostic model using logistic regression. Subsequently, the diagnostic ability of the model was evaluated using receiver operating characteristic curve (ROC) analysis.

### Institutional review board statement

The Ethics Committee of the Hainan Provincial Hospital of Traditional Chinese Medicine approved this study (reference number: 012/012). The test sample confirms that informed consent has been obtained from all subjects and/or their legal guardians. The supporting documents are attached to the related files.

### Compliance with ethics requirements

All procedures performed in studies involving human participants in this study conform to the ETHI-Cal standards of institutional and/or national research councils and the 1964 Declaration of Helsinki and its subsequent amendments or similar ethical standards.

## Results

### Data processing and analysis

**Figure 1:**
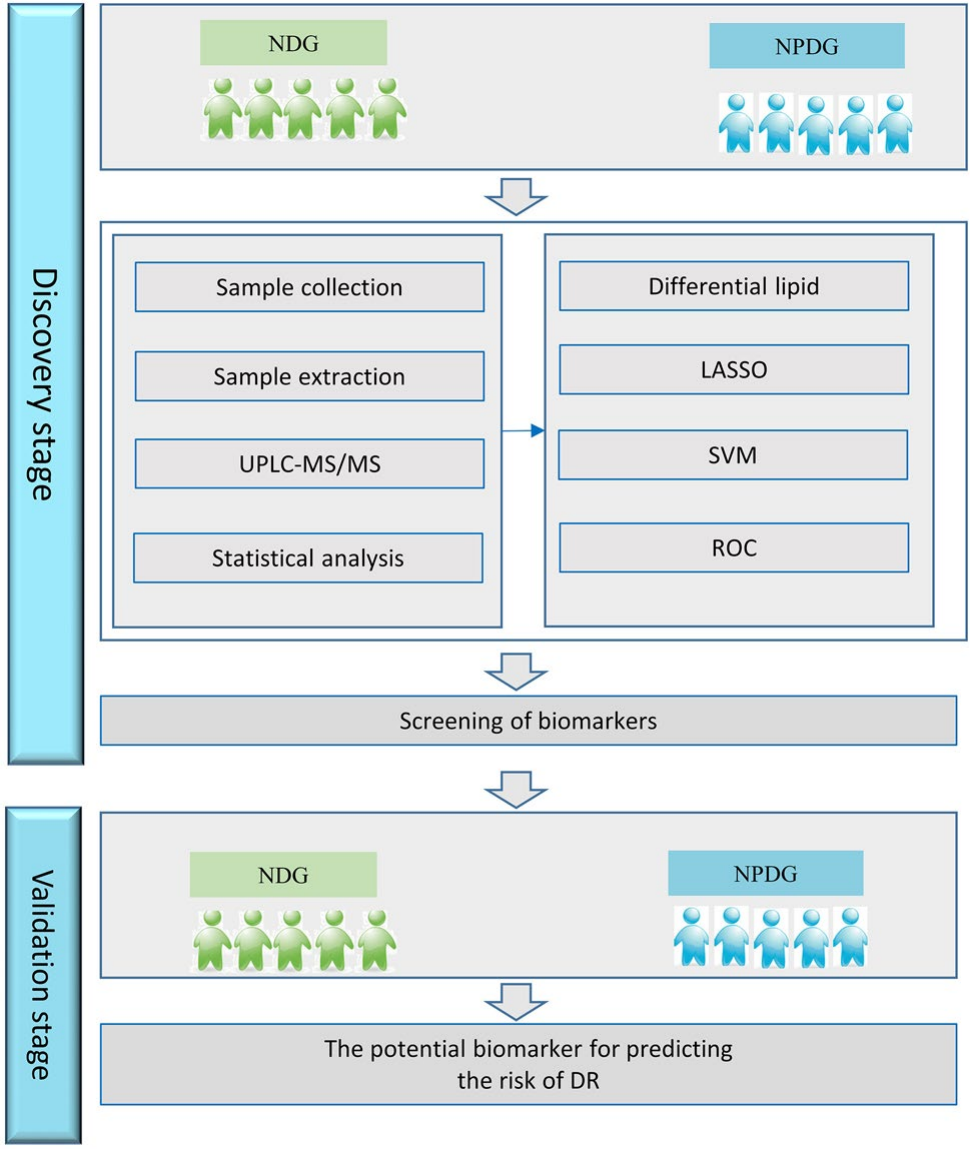
Workflow diagram.

This study employed a two-step analysis strategy, including discovery and validation steps. The workflow of this study is illustrated in Fig. 1. A total of 62 participants with T2DM, including 20 with NDR and 20 with NPDR, were included in the targeted lipidomics discovery cohort, and 22 (11 per group) were included in the independent external validation cohort.

**Figure 2:**
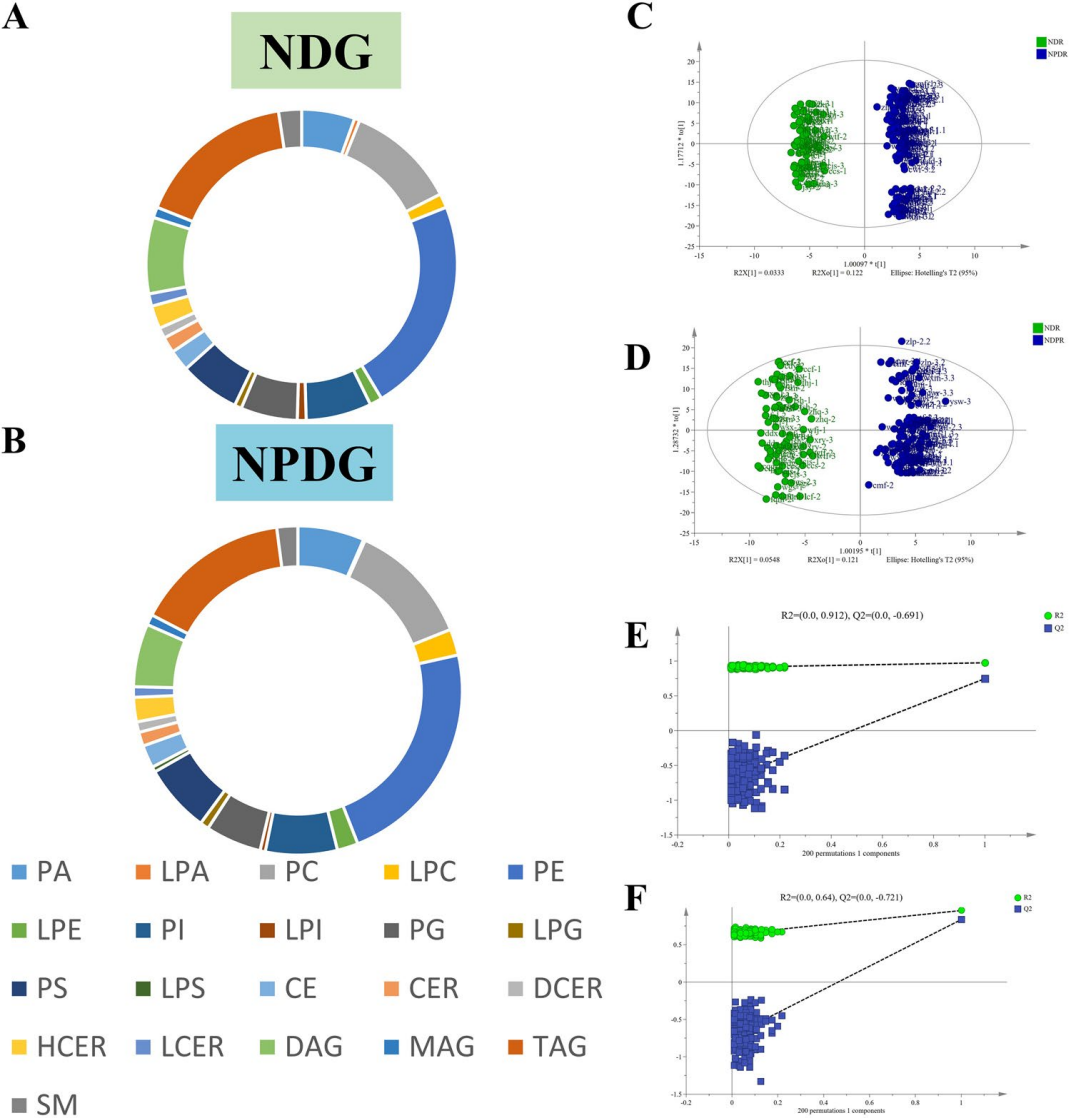
The total number of lipid subclasses detected by NDR (**A**) and NPDR (**B**), as well as the number of lipid compounds contained in each subclass; Orthogonal partial least squares discriminant analysis plot (OPLS-DA) of NDG and NPDG in negative ion mode: R2X = 0.503, R2Y = 0.977, Q2 = 0.748 (**C**, **E**) and OPLS-DA of NDG and NPDG in positive ion mode (OPLS-DA): R2X = 0.569, R2Y = 0.957, Q2 = 0.835(**D**, **F**). PA, phosphatidicacid; LPA, lysophosphatidic acid; PC, phosph-atidylcholine; LPC, lysophosphatidylcholine; PE, phosphatidylethanolamine; LPE, lysophosphatidylethanol-amine; PI,Phosphatidylinositol; LPI, lysophosphatidylinositol; PG, phosphatidylglycerol; LPG, lysophosphatidylgly-cerol; PS, phosphatidylserine; LPS, lysophosphatidylserine; CE, sterols; CER/LCER/DCER/HCER, ceramides; DAG, diglycerides; MAG, monoglyceride; TAG, triglycerides; SM, sphingomyelin.

In total, 934 lipid metabolites and 21 lipid subclasses were detected (Fig. 2A, B). Among the 21 lipid subclasses, the most common was phosphatidylethanolamines (PE; 131 species), followed by triglycerides (TAG) and Phosphatidylcholine (PC), with 90 and 62 species, respectively. Orthogonal partial least squares discriminant analysis (OPLS-DA) was performed on serum metabolomic data from the NDR and NPDR groups. The two-dimensional score plot of the OPLS-DA model in ESI+ and ESI− modes showed complete separation between the two groups, with clear intergroup differences and significant differences in the serum metabolome (Fig. 2C, D). The R2X, R2Y, and Q2 values of the OPLS-DA model in the positive ion mode were 0.569, 0.957, and 0.835, respectively, whereas those in the negative ion mode were 0.503, 0.977, and 0.748, respectively. The established data analysis model exhibited high data interpretation and prediction capabilities. To avoid overfitting, 200 permutation tests were performed, and the intercepts of Q2 on the y-axis were all less than zero (Fig. 2E, F), indicating that the model did not overfit.

### Screening, identification, and efficacy evaluation of biomarkers

**Figure 3:**
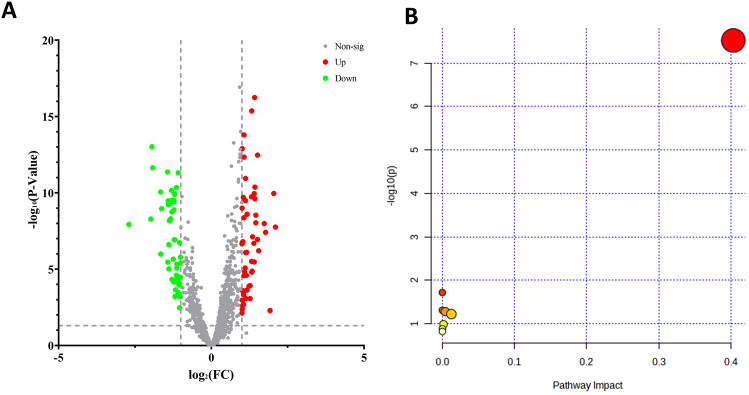
Volcanic map (**A**) and bubble map (**B**) of serum lipids of normal and patient differences. (**A**) Red, green, and gray in the figure represent upregulated, downregulated, and unexpressed differential lipid molecules, respectively. Differential lipid metabolites were defined as lipid metabolites with VIP ≥ 1, FC > 1.5, or FC < 0.66 and P < 0.05. (**B**) The vertical coordinates and color of the dots indicate the P values of the metabolic pathway analysis, with larger dots indicating greater differences in enriched metabolites and redder colors indicating more significant enrichment.

Based on the identified intergroup serum differential metabolome, with the screening criteria of P < 0.05, fold-change < 0.5 or > 2.0, and variable importance in projection (VIP) > 1.00, 102 differentially expressed lipid metabolites were identified between the NDR and NPDR groups. Among them, the expression levels of 54 were down-regulated and 48 were upregulated in the NPDR group compared to those in the NDR group (Fig. 3A). They metabolites included TAG, PE, phosphatidylglycerols, phosphatidic acids, phosphatidylinositols, diacylglycerols, and others. TAG accounted for 75% of the total composition of the 102 differentially expressed metabolites. Therefore, TAG metabolic disorder is an important abnormal feature in the sera of patients with DR. Subsequently, KEGG enrichment analysis was used to explore the potential functions of the differentially expressed lipid molecules. The results showed that they were mainly involved in glycerophospholipid metabolism, arachidonic acid metabolism, alpha-linolenic acid metabolism, GPI-anchor biosynthesis, glycerolipid metabolism, phosphatidylinositol signaling, arachidonic acid metabolism, and steroid biosynthesis (Fig. 3B).

### Construction of an early DR diagnosis model

**Figure 4:**
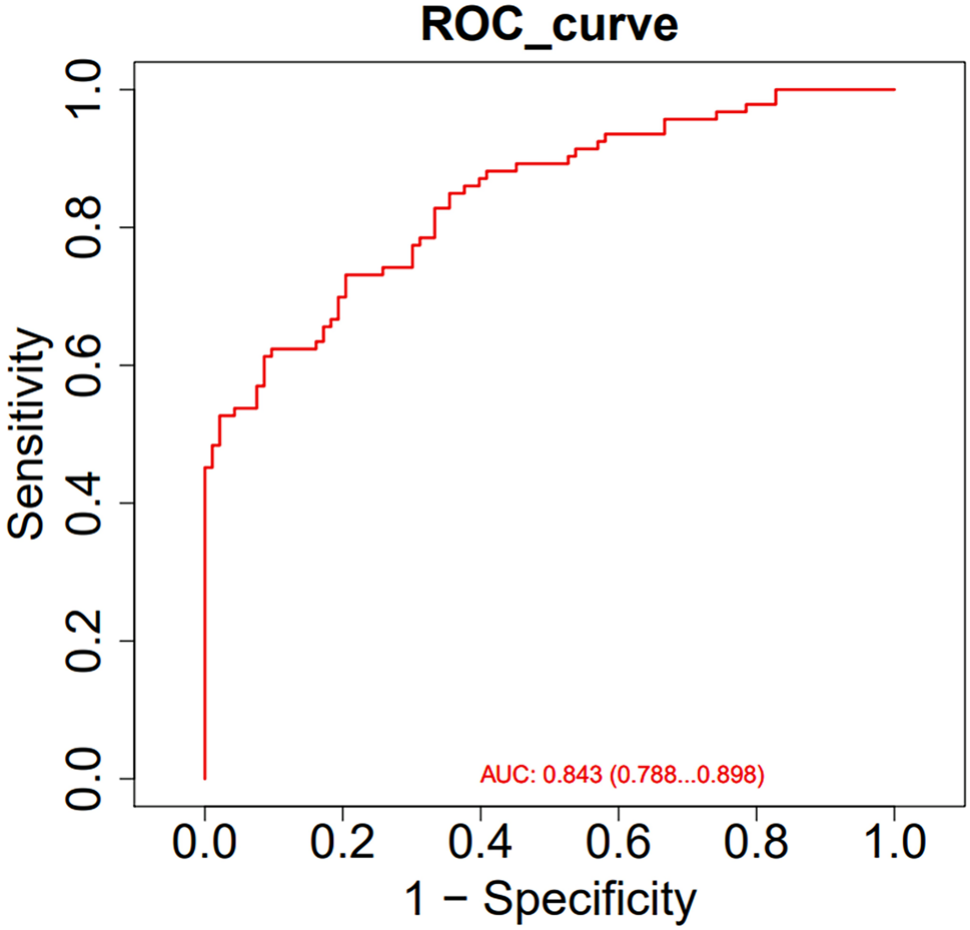
Subject performance profiles of potential biomarkers in serum. The AUC was the overall performance of four potentially risky lipid markers (TAG 58:2-FA18:1, 17:0 LPC, PA(18:0/18:2), and PS(18:1/22:6)).

Using LASSO and SVM regression analyses to screen concentrated lipid biomarkers, the NDR and NPDR groups were distinguished from each other. LASSO identified 34 lipid biomarkers with unique expression patterns in the NPDR group. The SVM-RFE algorithm was used to select the best features, and 14 lipid metabolites were identified as potential NDR biomarkers. To screen for relatively robust biomarkers, we intersected the two algorithms and obtained four lipid biomarkers: TAG58:2-FA18:1, 17:0 LPC, PA (18:0/18:2), and PS (18:1/22:6). The ROC curve analysis results showed that these had effective diagnostic performance in distinguishing patients with NDR from those with NPDR. The area under the curve (AUC) of these four lipid biomarkers were between 0.690 and 0.844. Combining them for diagnosis, the diagnostic model was effective in distinguishing between the NDR and NPDR groups, with an AUC exceeding 0.8 (Fig. 4).

## Discussion

DR is the most common and severe microvascular complication in patients with DM. It is the leading cause of low vision and blindness in the adult population, thereby significantly impairing the quality of life of several individuals. In this study, we used a comprehensive serum lipid metabolomics approach with UHPLC-MS/MS to analyze potential lipid biomarkers for the diagnosis of NPDR. Semi-quantitative detection of metabolites using internal standards improved the accuracy, reliability, and repeatability of the results. We used SVM to reduce the predicted variable set trained using LASSO regression to obtain convincing and robust biomarkers. We identified 102 differentially expressed lipid metabolites between patients with NPDR and NDR, with TAG accounting for 75%. We found that, when accounting for all variables, there were four lipid metabolites namely, TAG58:2-FA18:1, 17:0LPC, PA(18:0/18:2), and PS(18:1/22:6), with effective diagnostic performance that were able to distinguish NPDR from NDR. These four lipid metabolites may represent useful biomarkers for the early diagnosis of DR.

Jin et al., used nuclear magnetic resonance-based metabolomics to identify 25 differential metabolites in aqueous humor samples of older adult cataract patients with DM and with DR. These differential metabolites are mainly concentrated in the alanine, aspartic acid, and glutamate metabolic pathways. Although this study provides meaningful information, the specificity of the study subjects limits its scope for application^21^. Wang et al.^22^ used gas chromatography-time-of-flight mass spectrometry to identify 11 differential metabolites in vitreous and aqueous humor samples from patients with DR and patients without DM and elucidated the role of abnormal glucose metabolism, ascorbic acid–aldehyde metabolism, valine-leucine-isoleucine biosynthesis, and arginine–proline metabolism in the progression of DR. Although these studies were able to identify differential metabolites related to NDM and DR to some extent, they did not consider early screening indicators for DR. Due to the invasive nature of vitreous and aqueous humor sampling, as well as the small sample size, sample analysis became challenging. Lipidomics, in addition to providing information about individual lipids themselves, suggests the impact of specific lipid deficiencies on the organism^23^. Further, lipid metabolites have become promising potential biomarkers for various diseases; Huan et al.^24^ found that DR is highly correlated with lipid metabolism disorders. Rotbain et al., used LC–MS/MS targeted technology to analyze lipids in the serum of patients with stage 1 DM and significant albuminuria, and found that medium-sized unsaturated TAGs and lysophosphatidylcholines (LPCs) were more strongly associated with severe DR than the other lipids. LPC(16:1), TG(49:3), TG(50:1), and TG(50:2) were negatively correlated with DR and had indicative associations. However, these associations could not be replicated in the Analysis of Covariance models (ANCOVA) model, partly owing to the nonlinear trends of different lipids at different stages of DR. Yuan et al.^25^ identified and validated a novel biomarker combination of 12-hydroxyeicosatetraenoic acid (12-HETE) and 2-piperidone for distinguishing DR from early DR using non targeted GC-MSM, LC-MSM, and LC-MSL platforms, and tested its clinical practicality. Further, they found that the levels of most lipids showed a significant increase in DR compared to NDR, which is consistent with the results of our lipidomics analysis.

At present, early screening for DR is still a challenge, and many studies have reached different conclusions regarding this process. This may be due to the limited area covered by research sampling, as well as the complex effects of diet, climate, and lifestyle habits on metabolism. The PA (18:0/18:2) in the four lipids we obtained belongs to Phosphatidic Acid (PA), which can be phosphorylated by fatty acids and converted into phosphatidylinositol at last. Inositol is a sugar alcohol, proven to be related to the DR process in patients with type 2 diabetes^26^. Further, Curvic found that all metabolites significantly related to DR appear to be closely related to inositol. TAG58:2-FA18:1 belongs to TAG, which has beenproven to be an effective biomarker for dyslipidemia. Some treatment measures for dyslipidemia were found to be effective in retinopathy^4,19^. However, there are currently no relevant reports on PS (18:1/22:6) and DR. As expected, the four lipid biomarkers demonstrated good differentiation between NDR patients and NPDR patients in validation and whole group. This study has some limitations. Due to the limitations of sample location and quantity, further validation is needed. Moreover, there is no information available on lifestyle parameters at baseline, which may affect lipid analysis.

## Conclusions

Our findings indicate that metabolomic analysis can effectively identify diagnostic biomarkers for DR in patients with T2DM in China. Furthermore, our study presents four novel lipid biomarkers for NPDR and demonstrates that their combination in a diagnostic model exhibits excellent performance in differentiating between NDR and NPDR patients. Our findings provide a tool for the early diagnosis of DR and may assist in its prevention in the future.

## Data Availability

The data presented in this study are available on request from the corresponding author.
